# Extreme events in multilayer, interdependent complex networks and control

**DOI:** 10.1038/srep17277

**Published:** 2015-11-27

**Authors:** Yu-Zhong Chen, Zi-Gang Huang, Hai-Feng Zhang, Daniel Eisenberg, Thomas P. Seager, Ying-Cheng Lai

**Affiliations:** 1 School of Electrical, Computer and Energy Engineering, Arizona State University, Tempe, Arizona 85287, USA; 2 Institute of Computational Physics and Complex Systems, Lanzhou University, Lanzhou Gansu 730000, China; 3 School of Mathematical Science, Anhui University, Hefei 230039, China; 4 School of Sustainable Engineering and Built Environment, Arizona State University, Tempe, AZ 85287, USA; 5 Department of Physics, Arizona State University, Tempe, Arizona 85287, USA

## Abstract

We investigate the emergence of extreme events in interdependent networks. We introduce an inter-layer traffic resource competing mechanism to account for the limited capacity associated with distinct network layers. A striking finding is that, when the number of network layers and/or the overlap among the layers are increased, extreme events can emerge in a cascading manner on a global scale. Asymptotically, there are two stable absorption states: a state free of extreme events and a state of full of extreme events, and the transition between them is abrupt. Our results indicate that internal interactions in the multiplex system can yield qualitatively distinct phenomena associated with extreme events that do not occur for independent network layers. An implication is that, e.g., public resource competitions among different service providers can lead to a higher resource requirement than naively expected. We derive an analytical theory to understand the emergence of global-scale extreme events based on the concept of effective betweenness. We also articulate a cost-effective control scheme through increasing the capacity of very few hubs to suppress the cascading process of extreme events so as to protect the entire multi-layer infrastructure against global-scale breakdown.

Internal resource competitions are ubiquitous in complex dynamical systems, but relatively little attention has been paid to its impact on the dynamical evolution and resilience of the underlying interdependent, multilayer networked systems. Relevant situations include the airport and train-station networks of the public transportation system, the base station network of the cellular communication system, and the virtual networks based on software defined networks (SDNs). For example, in the airport network, different airlines cover different subsets of the airports across the whole country, and airlines operate in the same airport have to share and compete for the limited resources such as space and time. In the near future, different communication service providers may share base stations, generating potential competitions for bandwidth and processing capabilities of the based stations. The next generation of Internet may be built upon the framework of SDN, enabling one same physical server network to be virtually separated into multiple independent subnetworks with scalable sizes, each serving or being operated by a particular user without interfering with other subnetwork layers. In this case, the same server may carry the load generated by multiple layers (users), and its finite processing capacity is competed by the layers of virtual servers. All these call for a systematic study to understand the resilience of multilayer, interdependent networks subject to internal resource competitions. The goals of this paper are to develop a model capturing the key topological and dynamical features of the multilayer infrastructures incorporating inter-layer resource competitions, to study the extreme event dynamics from the standpoint of resilience, and to articulate control strategies to enhance the resilience against global-scale, catastrophic breakdown of the whole network system.

Extreme events associated with transportation dynamics on networks are intimately related to nodal flux fluctuations^1^,^2^,^3^,^4^,^5^,^6^,^7^. In previous models of single-layer transportation networks, extreme events tend to occur independently in space and time, which often have little effect on the system resilience^7^,^8^,^9^,^10^. However, for an interdependent network with multiple interacting layers, fluctuations can induce qualitatively distinct phenomena in the system that is intrinsically nonlinear. Recent works on multilayer networks have uncovered a rich variety of phenomena associated with network fragility and robustness^11^,^12^,^13^,^14^,^15^,^16^,^17^, diffusion and spreading processes^18^,^19^,^20^,^21^,^22^, game dynamics^23^,^24^,^25^,^26^, and synchronization^27^,^28^,^29^,^30^. Other related works range from redefining the basic structural measures to understanding the impacts of the multilayer nature of the network on dynamical processes. In spite of the previous efforts, to our knowledge there were no prior models of interdependent networks with multiple layers defined according to resource competitions, let alone any study of the extreme event dynamics in such networks.

In this paper, utilizing transportation as a prototypical dynamical process, we articulate an inter-layer traffic resource competing mechanism to characterize the situation where different network layers (e.g., corresponding to different social entities) coexist under limited capacities. A striking phenomenon is that, when the number of network layers and/or the overlap among layers are increased, extreme events can emerge in a cascading manner to trigger global-scale catastrophes, even when the capacity is capable of accommodating the same number of independent layers. We find that the system typically evolves into one of the two stable absorption states: a state free of extreme events and a state with frequent occurrence of extreme events, and the transition between the two states is abrupt in both time and parameter domains. The finding indicates that internal competitions in a multiplex network system can yield qualitatively distinct phenomena. An implication is that public resource competitions among different service providers can lead to higher resource requirement than anticipated. We derive an analytical theory to understand the emergence of global-scale extreme events based on the concept of effective betweenness that we specifically introduce to characterize and comprehend the extreme event dynamics on interdependent networks. We also articulate an efficient control scheme based on augmenting the capacity of very few hubs, which can dramatically suppress the cascading process of extreme events and protect the entire multi-layer infrastructure against global-scale breakdown.

## Results

**Figure 1:**
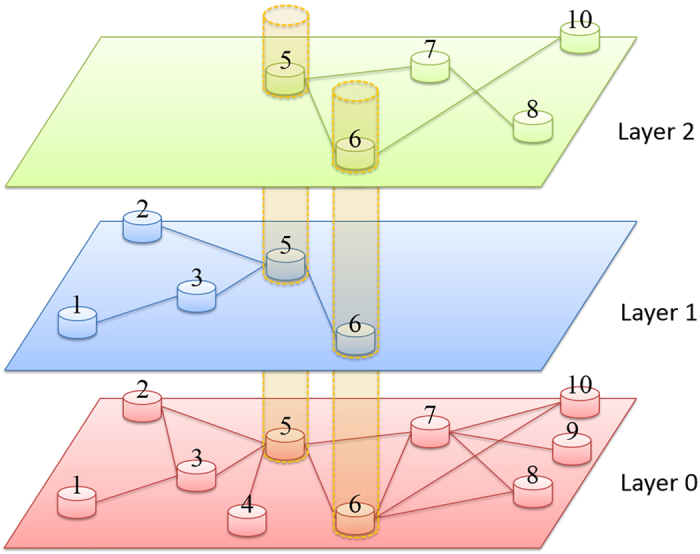
Schematic illustration of a multilayer, interdependent network subject to internal (inter-layer) resource competitions. Layers 1 (*G*_1_) and 2 (*G*_2_) are two different subsets of Layer 0 (*G*, or the global network). Note that Layer 0 is used to illustrate the process of model generation - it is not involved in the transportation dynamics. In this schematic example, *N* = 10 (number of node in Layer 0), *M* = 2 (the number of interdependent layers), and *P*_*m*_ = 0.5 (*m* = 1 and 2), where *P*_*m*_ ≡ *N*_*m*_/*N* with *N*_*m*_ being the number of nodes in layer *m*. Here, we have *N*_*m*_ = *N* ⋅ *P*_*m*_ = 5 and, correspondingly, the average nodal degrees of Layers 1 and 2 are half of that of Layer 0 (fluctuation exists due to randomness). The combination of Layers 1 and 2 forms a multilayer interdependent network. Nodes 5 and 6 exist in both Layers 1 and 2, and thus, the two layers would compete for resources through these two nodes. Nodes 4 and 9 exist in neither of the two layers. Each of the other nodes exists in only one of the two layers (see **Methods**).

Our model is motivated by the setting of a large infrastructure, which we regard as a complex network *G* of *N* nodes. Among the *M* service providers, each provides packet transportation service on a subnetwork *G*_*m*_(1 ≤ *m* ≤ *M*) of *N*_*m*_(*N*_*m*_ ≤ *N*) nodes connected via the links of *G*. The entire network *G* thus has *M* layers, each being the subnetwork operated by one service provider, as shown schematically in Fig. 1. For convenience, we call *G* the global network. Packets belonging to layer *G*_*m*_ have their origins and destinations solely within *G*_*m*_: they can only be transported within *G*_*m*_ through its nodes and links, not to any other layer. Different layers can share common nodes, and the number of layers sharing node *i* is denoted as *M*_*i*_.

The load of node *i* in *G* is defined as the total number of packets from all the layers containing node *i*, i.e., 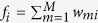, where *w*_*mi*_ is the flux in layer *m*, among which there are *M*_*i*_ nonzero values. If *f*_*i*_ reaches the capacity *C*_*i*_ of node *i*, we say that an extreme event has occurred on this node. The capacity *C*_*i*_ can be written as^7^


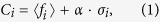


where 〈 *f*_*i*_〉 is the average load, *σ*_*i*_ is the standard-deviation of *f*_*i*_, and *α* > 1 is the capacity parameter. Following ref. 7, we set *α* = 4 (somewhat arbitrarily) in our study. In fact, variation in *α*, insofar as it is larger than unity, will not affect the results qualitatively. This setting allows the capacity of node *i* to be adjusted according to the number of layers *M*_*i*_ sharing it see **Methods**. For simplicity, we assume that all layers have the same nodal coverage, i.e., *P*_*m*_ = *N*_*m*_/*N*, where *P*_*m*_ = 1 corresponds to the special case that every layer is identical to the whole network, i.e., *G*_*m*_ = *G*.

The rules for packet transport/delivery are as follows. Within each layer, at each time step, 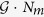 new packets are generated, where 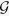 is the packet generation rate. The newly generated packets have randomly assigned destinations and are originated from randomly selected nodes that are free of any extreme events at the time. On a node, each previously generated packet is transported to a neighbor of the node along the shortest path towards the packet’s destination. If no extreme event occurs on the target neighbor at this time, the movement can be completed. If, however, there is an extreme event on the node that the packet is supposed to move into, the packet is transported to a randomly chosen neighbor with no extreme event, provided that such a neighbor exists. If all the neighbors are currently having extreme events, the packet will remain at the original node.

### Global extreme events and two absorption states

**Figure 2:**
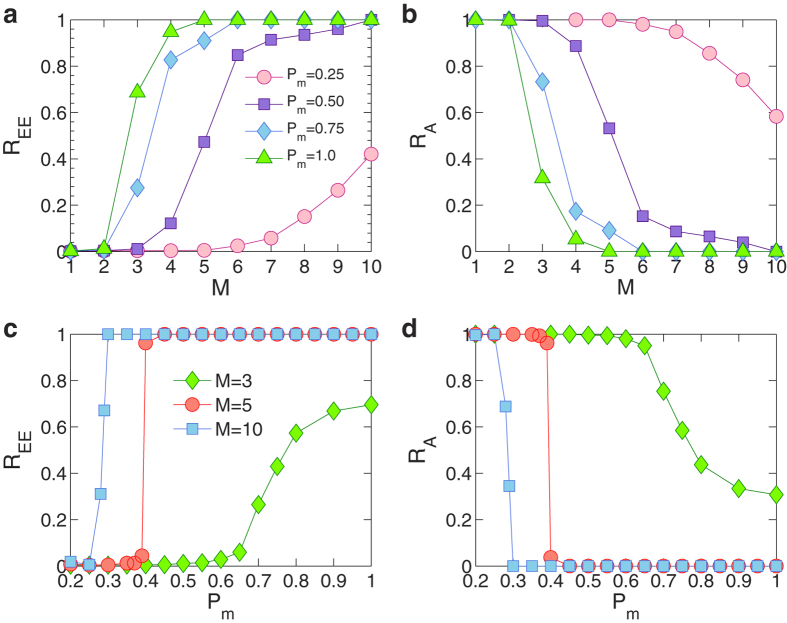
Extreme event occurrence and packet arrival rates. (**a**) Extreme event occurrence rate *R*_EE_ and (**b**) packet arrival rate *R*_A_ versus the number of layers, *M*, for systems with *P*_*m*_ = 0.25 (circles), 0.5 (squares), 0.75 (diamonds), and 1 (triangles), respectively. (**c**) *R*_EE_ and (**d**) *R*_A_ versus *P*_*m*_ for *M* = 3 (diamonds), 5 (circles), and 10 (squares), respectively. The results are obtained through 20 simulation realizations for each of the 10 network realizations. Each network has the average degree 〈*k*〉 = 4 and size *N* = 1000. Each realization runs for 500 time steps. The quantities *R*_EE_ and *R*_A_ are averaged over the last 300 steps (typically, the system evolves into equilibrium within 200 time steps).

To quantify the transportation dynamics on the multilayer network, we use two quantities: (1) extreme event occurrence rate *R*_EE_, defined as the fraction of nodes at which extreme events occur at each time step, and (2) packet arrival rate *R*_A_ defined as the ratio between the numbers of packets arriving and newly generated. Figure 2 shows the behaviors of *R*_EE_ and *R*_A_ for various numbers of layers and nodal coverage values. As shown in Fig. 2(a,b), *R*_EE_ for a single layer network (*M* = 1) is close to 0%, and *R*_A_ is 100%, implying that the capacity of a single layer network is sufficient to handle all the load in the network. As the number of layers is increased, the capacity is increased accordingly. If the layers are independent of each other, the total capacity can accommodate all the load from all layers, ruling out extreme events. However, when the layers are interdependent, there is a dramatic increase in *R*_EE_, indicating much higher total load generation due to internal resource competitions among the layers. The competitions drive the load of many nodes to their capacities, finally leading to the occurrence of extreme events on a global scale, i.e., *R*_EE_ = 100% and *R*_A_ = 0. Further computations reveal another phenomenon: after the system evolves into an equilibrium, only two types of steady absorption states can arise. They are (1) a *free state* nearly free of extreme events (*R*_EE_ ≳ 0), and (2) a *catastrophic state* in which every single node has an extreme event. The surprising feature is that there are no stable intermediate states in between the two cases. In fact, we find numerically that any intermediate state is transient in the sense that it must eventually evolve into one of these two absorption states. The value of *R*_EE_ thus represents the probability of emergence of the catastrophic state.

Note that, the value of *R*_EE_ + *R*_A_ is approximately unity. The reason is that the nodes with extreme events occurring on them will not accept any newly arrived packets. As a result, these packets can only reach other nodes in the network at time time, the portion of which is 1 − *R*_EE_. Since the packet destinations are uniform, the fraction of the packets that can arrive at their destinations is approximately 1 − *R*_EE_. Before the final equilibrium is achieved, at each time step, there is no guarantee that the sum of the extreme event occurring rate and the packet arrival rate is exactly unity, due to asynchronized updating and randomness. If the system reaches a catastrophic, global extreme-event state, we have *R*_EE_ = 1 and *R*_A_ = 0 so that *R*_EE_ + *R*_A_ = 1 holds exactly. Otherwise, in a free state where no extreme events occur, we have *R*_EE_ ≈ 0 and *R*_A_ ≈ 1, so *R*_EE_ + *R*_A_ ≈ 1.

### Abrupt transition

As shown in Fig. 2(a,b), the curves corresponding to higher *P*_*m*_ values have systematically higher *R*_EE_ values, indicating that *P*_*m*_ has an effect on the probability of the catastrophic state. Figure 2(c,d) are further results of how *R*_EE_ and *R*_A_ vary with *P*_*m*_ when the number of layers (*M*) is fixed. A higher value of *P*_*m*_ means that it is more likely for two different layers to share common nodes, signifying a higher degree of interdependence with more severe internal competitions. As a result, *R*_EE_ (or *R*_A_) increases (or decreases) monotonically with *P*_*m*_. A striking behavior occurs for relatively large values of *M*, where *R*_EE_ (*R*_A_) exhibits an abrupt increase (decrease) as *P*_*m*_ passes through a critical point, giving rise to an abrupt transition. In fact, about the transition point, the probability for the system to evolve into a catastrophic state can exhibit a dramatic change, i.e., from 0 to 100% or vice versa, meaning that an arbitrarily small change in *P*_*m*_ can drive the system into catastrophe. The value of *P*_*m*_ thus has a critical impact on the emergence of extreme events on the global scale. The implication is that, for an infrastructure consisting of multiple, interdependent layers, its resilience against catastrophe can exhibit a sensitive dependence on the system parameter, such as *P*_*m*_, whose critical value depends on the number of layers. In the design of infrastructural systems, it is then necessary to construct a detailed multilayer network model to estimate the critical parameter region of the abrupt transition, and the choices of the parameters should be such that they are far away from the critical region with large margins.

### Time evolution and packet lifetime

**Figure 3:**
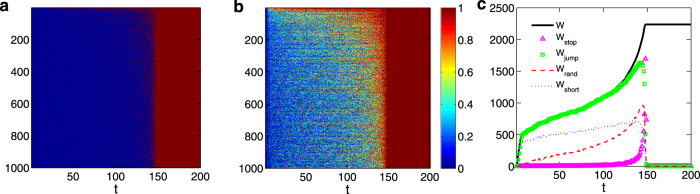
System’s time evolution towards a catastrophic state. For a multilayer network of *N* = 1000 nodes, *M* = 3 layers, and *P*_*m*_ = 1, (**a**) transition between a free (blue) and a catastrophic states (red) in time for all nodes. (**b**) The ratio of node *i*’s load *f*_*i*_ to its capacity *C*_*i*_ versus time for all nodes. (**c**) Time traces of the total number of packets in the layer (denoted as *W*, thick solid line), of the number of “stuck” packets due to the surrounding extreme events (*W*_stop_, triangles), and of the number of movable packets (*W*_jump_, squares). Among the *W*_jump_ movable packets, the number of those that effectively execute random walks (*W*_rand_, red dashed line) and the number of the remaining packets moving along their respective shortest paths (*W*_short_, blue dot line) are also plotted versus time.

In order to understand the emergence of extreme events at the global scale, we investigate the time evolution of various dynamical quantities. Figure 3(a) shows the time evolution of the states of all nodes in the network, where at any time, each node can be in one of the two possible states: free (blue) and catastrophic (red). Figure 3(b) shows the relative load of each node, *f*_*i*_/*C*_*i*_, versus time. In both panels, a sudden transition can be seen immediately before *t* = 150, at which the entire population of nodes is taken over by extreme events almost simultaneously. After the transition, transportation dynamics in the entire system is completely stalled.

If no extreme event occurs throughout the system, each packet would follow the shortest path towards its destination without any delay so that its lifetime *τ* equals the length *L* of the path. In equilibrium, the expected total number of packets accommodated in layer *m* is given by (see **Methods**)


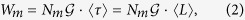


where 〈*τ*〉 is the average packet lifetime and 〈*L*〉 is the average shortest path length of the layer. An extreme event occurring at the packet’s next target node along the shortest path is most likely to increase the lifetime *τ* by causing the packet to take one step onto a randomly selected neighbor of the current node (carrier), because the probability is small for such a neighbor to locate along another shortest path of the same length. In the case where extreme events occur on all current carrier’s neighbors, the packet will stop moving and wait until at least one of the neighboring nodes becomes free of extreme events. This scenario will cause *τ* to increase. Figure 3(c) shows some quantities characterizing packet movements in one layer versus time as the system evolves from a free state into a globally catastrophic state. At each time step, the total number of packets in the layer, *W*, equals the sum of *W*_jump_, the number of moved packets, regardless of whether the packets are along the shortest path, and *W*_stop_, the number of stuck packets due to the extreme events in the neighborhood. The quantity *W*_jump_ is equal to the sum of *W*_short_, the number of packets that have moved along their corresponding shortest paths, and *W*_rand_, the number of packets that are stuck through random walks. Based on these considerations, we can write


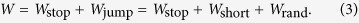


Until a global catastrophe takes place, *W*_stop_ is close to zero, *W*_short_ increases slightly but *W*_rand_ increases much faster with time. The behaviors of *W*_jump_ and consequently *W* are thus dominated by that of *W*_rand_, demonstrating the key role played by *W*_rand_: a few extreme events lead to random walks that lead to an increase in 〈*τ*〉 and consequently to an increase in *W*. The larger number of packets in turn cause the total load to increase, enhancing the probability of extreme events, which further generate more random walks. This self-stimulating, positive feedback type of mechanism is responsible for driving the whole system into the catastrophic phase.

For a single-layer network, a catastrophic phase cannot arise. In this case, 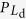, the fraction of packets that are *L*_d_ steps away from their destinations, are constant in equilibrium. Assuming that packets corresponding to each *L*_d_ value have the same probability *η* to be driven into random walks, we can solve both 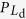 and *η* analytically by using the principle of detailed balance from statistical physics (see **Methods**).

### Positive feedback loop

Due to the internal resource competitions, the following process emerges: Inter-layer at a time step, the resource requirement in layer *m* at node *i* is high, and a substantial portion of node *i*’s capacity is devoted to layer *m*. As a result, other layers sharing node *i* will have to redistribute their load onto *i*’s neighbors, squeezing the capacity available for layer *m* of these neighbors and leading to reduction of their load back onto node *i* in layer *m*, and so on. This mechanism can trigger a cascading process and finally drive the system into a global-scale catastrophe. The cascading process is more likely to take place in systems with more layers.

### Betweenness and effective betweenness

It is difficult to develop a rigorous mathematical theory to fully grasp the mechanism of the cascading process. Our aim is to provide a physical understanding. In fact, the probability for an extreme event to occur on a node can be *quantitatively* analyzed through estimation of the nodal flux distribution function using the concept of nodal betweenness^31^, denoted as *B*(*i*). Previous works^2^,^5^,^7^,^10^,^32^ established that, for a single-layer transportation network, under random routing the probability for a node to be visited is proportional to its degree, but for the shortest-path routing scheme, the probability is proportional to the number of shortest paths through the node. Motivated by these considerations, we hypothesize that the probability for node *i* to be visited is determined by the following normalized betweenness:


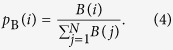


Under the shortest path routing scheme, the transportation dynamics can be regarded as a random-walk process with nodal visiting probability *p*_B_(*i*). If packets are uniformly distributed on various paths of different length, the betweenness centrality scales with the nodal degree in a power-law fashion^31^,^33^:


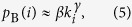


where *β* is a normalization constant and *γ* is the power-law scaling exponent (both parameters can be determined numerically). This relationship holds for most degree values (see **Methods**), providing a basis for our physical analysis.

Consider now a multilayer network. For layer *m*, the probability density function (PDF) of the flux *w*_*mi*_ through node *i* is binomial:


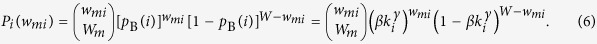


For simplicity, we set *P*_*m*_ = 1 so that all layers are identical. In this case, we can replace *W*_*m*_ with 〈*W*〉 so that the number of layers sharing node *i* is simply *M*_*i*_ = *M*. The total load on node *i* from the *M* layers, 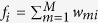, can then be written as a sum of *M* binomial random variables with identical distributions as given by Eq. (6). During the dynamical evolution, interdependence among the *M* random variables is taken into account through the inequality *f*_*i*_ ≤ *C*_*i*_. It is useful to calculate the probability of *f*_*i*_ = *C*_*i*_, the criterion for an extreme event to occur on node *i*. If we set the system free by removing the capacity bound of every node and allowing *f*_*i*_ to increase indefinitely, the probability will essentially be given by *P*( *f*_*i*_ ≥ *C*_*i*_), where *f*_*i*_ is the sum of *M* independent, identically distributed binomial random variables. The PDF of *f*_*i*_ is then given by


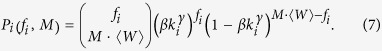


**Figure 4:**
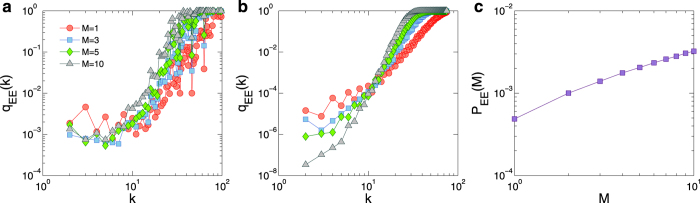
Numerically obtained and theoretical predicted probabilities of extreme events. For *M* = 1 (circles), 3 (squares), 5 (diamonds), and 10 (triangles), (**a**) numerically obtained probability *q*_EE_(*k*) for an extreme event to occur on a node of degree *k* and (**b**) theoretical prediction based on the concept of betweenness. (**c**) For *P*_*m*_ = 1, the probability of extreme events versus *M*, where the degree distribution function *P*(*k*) used in the calculation of *q*_EE_ is 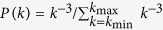 for *k*_min_ = 2 and *k*_max_ = 75 (typical values from network realizations). In fact, small variations in the values of *k*_min_ and *k*_max_ do not affect the main features of the results.

The probability for an extreme extent to occur on a node of degree *k* is


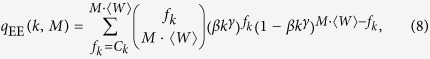


where *f*_*k*_ and *C*_*k*_ denote the load and the capacity of a node of degree *k*, respectively. As shown in Fig. 4(a,b), this analysis captures the qualitative behavior of the system from simulation. We further see that, for nodes of relatively high degrees, *q*_EE_(*k*) for high values of *M* (or *k*) is generally larger than those for lower values of *M* (or *k*), revealing that extreme events tend to occur on nodes shared by more layers and of larger degrees. This result should be contrasted to the case of single layer networks, where extreme events tend to occur more on small degree nodes, e.g., under the random routing scheme^7^. Thus, in multilayer networks, the hub nodes play a crucial role in generating extreme events. Based on this, we can write the probability for an extreme event to occur in a *M*-layer system as


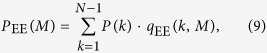


where *P*(*k*) is the degree distribution of the entire network. Figure 4(c) reveals a monotonically positive correlation between *P*_EE_(*M*) and *M*, providing a qualitative explanation for the more frequent occurrence of extreme events in systems with more layers.

**Figure 5:**
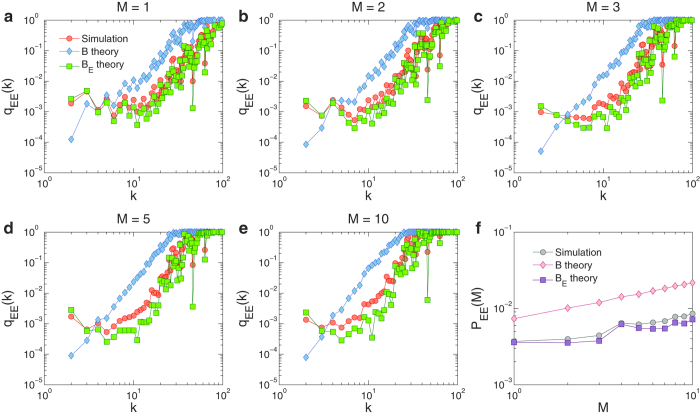
Probability of extreme events calculated from our theory of effective betweenness. (**a**–**e**) The probability *q*_EE_(*k*) for an extreme event to occur on a node of degree *k* obtained from simulation (circles), the theory of betweenness (diamonds), and the theory of effective betweenness (squares) for *M* = 1, 2, 3, 5, and 10, respectively. In (**f**), the extreme-event probability versus *M* is shown, obtained from simulation (circles), the theory of betweenness (diamonds), and the theory of effective betweenness (squares), for *M* = 10 and *P*_*m*_ = 1. The degree distribution function *P*(*k*) used to calculate *q*_EE_ is obtained using 10 network realizations. The betweenness and effective betweenness for each node of degree value *k* are calculated from the corresponding network topology and averaged over all such nodes among the 10 network realizations.

If we calculate *p*_B_(*i*) using the actual betweenness values for all the individual nodes and then averaging the nodes of the same degree (which can be done based on the topological information of the network) instead of using the approximation Eq. (5), we find systematical deviation in *q*_EE_(*k*) as given by theory from that through simulation, as shown in Fig. 5(a–e). Empirically, we are able to identify a quantity to replace the betweenness centrality in determining *q*_EE_(*k*) and *P*_EE_(*M*). We name it *effective betweenness*, defined for node *i* as


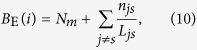


where *L*_*js*_ is the length of a shortest path through *i* between the origin *j* and the destination *s* (*j* ≠ *s*), and *n*_*js*_ is the number of such paths. While a rigorous justification for the effective betweenness is difficult, it gives a better agreement between theory and numerics. An intuitive understanding is the following. For *j* ≠ *s*, i.e., *L*_*js*_ > 0, since the lengths of these shortest paths are proportional to the lifetime of the packets, the packet assigned to a longer path *L*_*js*_ has a longer lifetime. Along the path, the packet occupies each node once, thereby contributing less to the visiting probability of a given node in each time step. The term *N*_*m*_ counts for the contribution from the case of *j* = *s*, i.e., *L*_*js*_ = 0.

Using the concept of effective betweenness, we obtain the probability of a node to be visited as


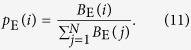


More justifications for the use of the effective betweenness can be obtained through simulations (see **Methods**).

In a similar manner, we can calculate *q*_EE_(*k*) and *P*_EE_(*M*) through *p*_E_(*i*) instead of *p*_B_(*i*). Figure 5(a–e) show, for a number of different settings, good agreement between the predicted values of *q*_EE_(*k*) with the numerical values. In fact, the predicted values of *P*_EE_(*M*) through *B*_E_ also match the simulation results very well, as compared with those through *B*, as shown in Fig. 5(f). These results indicate that the effective betweenness is a key quantity characterizing the transportation dynamics on multilayer networks subject to inter-layer competitions under the standard shortest path routing protocol. Utilizing this concept, the transportation dynamics can effectively be mapped into a binary stochastic process for further theoretical development.

### Control strategies to suppress extreme events

From Fig. 2(c,d), we see that a small increase in the parameter *P*_*m*_, when it is near the critical point, can lead to a transition between the free and catastrophic states. A straightforward control strategy is then to reduce the value of *P*_*m*_. Since extreme events tend to occur on nodes shared by many layers, controlled reduction of the overlap between the layers can also suppress extreme events. However, these naive methods will not be effective when *P*_*m*_ has well passed the critical point.

**Figure 6:**
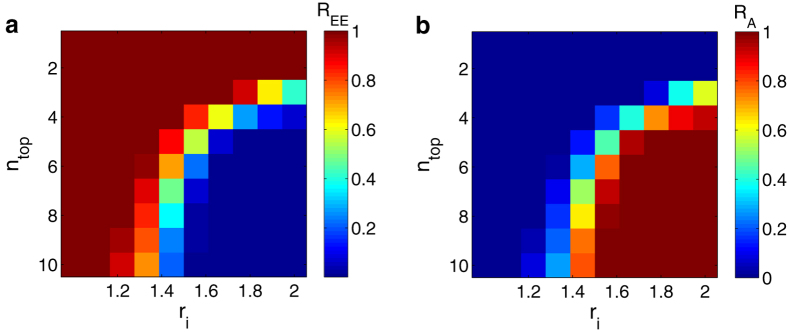
Occurrence rate of extreme events and packet arrival rate under control. In the control parameter space, *n*_top_ denotes the number of top-degree hubs whose capacities are augmented, and *r*_i_ is the ratio of the enhanced capacity to the original capacity. The extreme event occurrence rate *R*_EE_ and the packet arrival rate *R*_A_ in the parameter space are shown, respectively in (**a, b**). The simulation parameters are the same as in Fig. 2.

We focus on the case where *P*_*m*_, by design, exceeds its critical value by a large amount. One can increase the capacity of each node to prevent the occurrence of extreme events at a global scale, but this may be costly. According to Fig. 4(a), despite small fluctuations, extreme events take place on large degree nodes with a higher probability. A practical strategy is then to selectively enhance the capacities of the hub nodes. Figure 6(a,b) show, respectively, *R*_EE_ and *R*_A_ when the capacities of the top *n*_top_ nodes (ranked by degree) in the network are multiplied by the factor *r*_i_ > 1. There exists a region in the parameter space that is completely free of extreme events, i.e., *R*_EE_ ≈ 0 and *R*_A_ ≈ 1, with a clear boundary separating this region from the catastrophic regions. We see that neither too small values of *r*_i_ nor small values of *n*_top_ can inhibit extreme events, implying that the extreme events occurring at a small set of hub nodes form a positive feedback loop through mutual stimulation and accordingly trigger global-scale cascading processes. We regard these hubs as constituting an *extreme-event core* (EE core), since they serve as the source of the global catastrophe. We see that extreme events can be effectively eliminated by providing reasonably more resources to nodes in the EE core. For example, solely increasing the capacities of the top 5 hubs (5 out of 1000, less than 1%) by 1.7 times can make the entire network system immune to any global cascade. The enhanced capacity is in fact insignificant comparing with the total capacity of the system, but the targeted capacity enhancement can improve the network resilience disproportionally by drastically reducing the probability of extreme-event cascade. For a multilayer network system of infrastructure, it is thus important to identify the EE core and to assign larger capacities to the nodes in the core.

## Discussion

The ubiquity of resource competitions in complex infrastructure systems motivates us to articulate and study a class of interdependent complex networks in which a set of nodes representing, e.g., public service facilities in a large infrastructure, are shared by different layers that correspond to, e.g., different service providers. The set of shared nodes have relatively large capacities. We find that internal competitions for common resources have a dramatic and sometimes devastating effect on the network transportation dynamics. In particular, as the number of network layers is increased, extreme events in which the delivering capabilities of certain nodes in the network are essentially depleted can occur in a cascading manner, leading to a catastrophic occurrence of such events on a global scale. This should be contrasted to the case of total absence of extreme events in systems with the same nodal capacities but without internal competitions (so that the layers are independent). A striking phenomenon is that, there are two distinct possible asymptotic states for the system: (1) a state free of extreme events and (2) a state completely dominated by extreme events. Varying one of the two key structural parameters, the number of layers and the nodal coverage rate, an abrupt transition can occur between the two states, meaning that the system can change abruptly from one state to another as a control parameter passes through a critical point. We develop a physical theory to understand the dynamics of extreme events based on a newly defined topological property, the effective betweenness, and the empirical scaling law for betweenness centrality in general. To suppress extreme events and enhance the resilience of the system against global-scale breakdown, we propose and test an effective and low-cost control strategy, the articulation of which benefits from the finding of an *extreme events core* formed by a small number of hub nodes, which plays a critical role in “spreading” the extreme events. When the capacities of the nodes in the core are selectively augmented, the network’s ability to resist large-scale extreme events can be enhanced significantly.

Our findings have potential applications in gaining insights into the resilience of large scale infrastructural systems that are typically composed of many layers with shared public service facilities. Our results indicate that competitions for public resources can lead to catastrophic behaviors, and control is necessary to make the system resilient to large scale failures. Generally, control of extreme events in interdependent networked system is an important and challenging problem, and we hope our work to stimulate further efforts.

## Methods

### Model details

For a given network *G* (Layer 0, shown in Fig. 1), each of the *M* subnetwork layers is generated via the following procedure: (1) randomly select a node in *G*; (2) randomly select its *P*_*m*_*k* neighbors; (3) for each selected neighbor, do (2) and repeat the step for all newly selected nodes until the total number of selected nodes reaches *P*_*m*_*N*. This procedure fixes the average degree of each layer to be *P*_*m*_〈*k*〉. To be concrete, we use the Barabasi-Albert (BA) type of scale-free topology^34^ for network *G*. The power-law exponent of the degree distribution for each layer is approximately the same as that for the entire network *G*.

Due to randomness in the packet transportation process, the flux of node *i* in layer *m*, *w*_*mi*_, is a random variable. Accordingly, *f*_*i*_ is the sum of *M* random variables (with *M*_*i*_ of those being nonzero). If different layers are independent and packet transportation process can be described as a random walk, we have


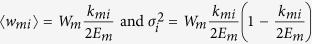


where *W*_*m*_ is the total number of packets in layer *m*, *k*_*mi*_ is the degree of node *i* in layer *m*, and *E*_*m*_ is the total number of links in layer *m*. Hence, we have


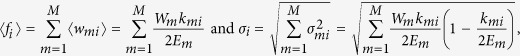


where *W*_*m*_ can be determined numerically through a single layer simulation or be calculated analytically from Eq. (2).

Numerical simulations of the dynamical process of transportation are carried out, as follows. Initially no packets exist in the system. At each time step, 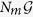 newly generated packets with random destinations are placed on randomly selected nodes that are currently *free of extreme events*. Any node with load equal to its capacity is not allowed to accept any packet from its neighbors, but the packets on the node can still leave the node, if at least one of its neighbors is not fully loaded. Asynchronous updating scheme is adopted in the simulation to ensure that no node has load higher than its capacity and also for the reason that synchronization in large infrastructure systems is not always realistic and necessary. (In fact, many real systems are strongly asynchronous, e.g., the planes or trains usually enter an airport or a train station one after another). The number of newly generated packets in the network is determined by the packet generation rate 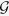 and the layer population *N*_*m*_ = *P*_*m*_*N*. Without loss of generality, we treat *P*_*m*_ as a tunable parameter while keeping 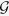 fixed. Variations in 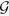 do not affect the results qualitatively. The packet generation rate is set to be 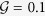 in our study.

### Total number of packets in a layer

In an equilibrium state without extreme events on a global scale, each packet can move freely along the shortest path towards its destination so that its lifetime *τ* is approximately equal to the length *L* of the path. Since *W*_*m*_(*t*) includes *W*_*m*_(*t* − 1) and the newly generated 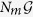 packets with the number of arrivals subtracted off, we can derive the expression of *W*_*m*_ step by step. In particular, starting from *t* = 1, we have


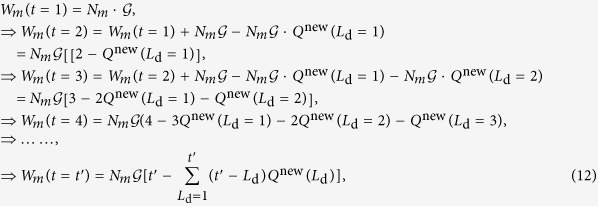


where *Q*^new^ (*L*_d_) denotes the stable fraction of the newly generated packets that are *L*_d_ steps away from their destinations. Apparently, if 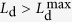, i.e., if *L*_d_ is larger than the maximum shortest path length 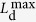 in the focal layer, we have *Q*^new^ (*L*_d_) = 0 and


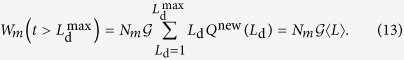


The relaxation time for the system to evolve into the stable equilibrium state is given by 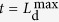. We obtain *W*_*m*_(*t* → ∞), as in Eq. (2). However, when extreme events take place in the system, the packet lifetime *τ* can assume values much larger than the path length *L*. In this case, we have 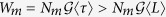.

### Effect of rerouting via random walk

For a single-layer network, we can develop a theory to explain the qualitative behavior of the transportation dynamics. In an equilibrium state, the probability *Q*(*L*_d_) for a packet to be *L*_d_ steps away from its destination satisfies the condition of detailed balance. For simplicity, we assume that each packet commits one step random walks with probability *η*, regardless of the distance from its destination. A walk makes a packet one more step away from its destination (which is numerically observed with high probability). Thus, at time *t*, packets corresponding to *L*_d_ constitute (1) the packets corresponding to *L*_d_ + 1 at time *t* − 1 and moved along the shortest path towards their destinations at time *t*, (2) the packets corresponding to *L*_d_ − 1 at time *t* − 1 and committing random walks at time *t*, and (3) the newly generated packets corresponding to *L*_d_. Consequently, for 
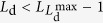
, we have


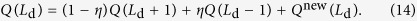


Since, in a typical case, any movement of the packets corresponding to 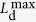 makes their distances from the destinations smaller, packets for 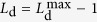 include all these that are 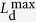 steps away from their destinations. We thus have


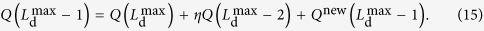


As boundary conditions, for packets corresponding to 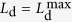, we have


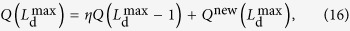


and for packets corresponding to *L*_d_ = 1, we have


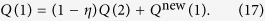


Consider the case where 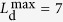 (a typical case in our study). We obtain a complete equation set for solving *Q*(*L*_d_), as follows:


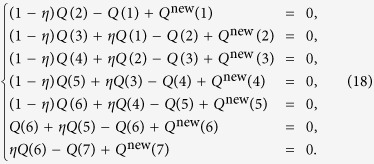


It can be written in the matrix form


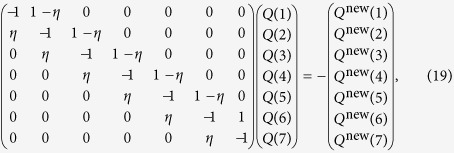


where


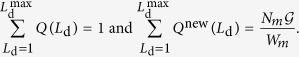


**Figure 7:**
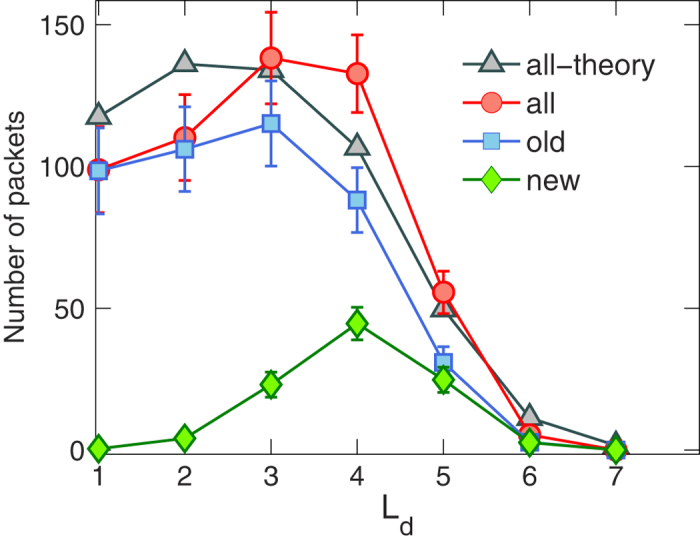
Number of packets *L*_d_ steps away from their destinations. For a single layer network of size *N* = 1000, the number of packets in an equilibrium state that are *L*_d_ steps away from their respective destinations from theory (triangles) and simulation (circles). The total number of packets that the system can accommodate in the equilibrium state is about *W* = 558, which includes the newly generated packets within the current time step (diamonds) and all the remaining old packets (squares).

This set of equations can be solved numerically. When the network topology is fixed, *Q*^new^ (*L*_d_) follows a constant distribution, which can be obtained from simulation. While each value of *η* generates a set of *Q* (*L*_d_) values, a proper value makes the sum of *Q* (*L*_d_)’s unity. In our calculation, as *η* is increased from 0 to 1, we find that the sum of the *Q *(*L*_d_) increases monotonically and, hence, only the *η* values that make 
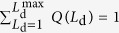
 provide a solution. Figure 7 shows the theoretical solution of the *Q* (*L*_d_) distribution, which qualitatively agrees with the simulation result.

### Effective betweenness calculation and flux distribution

**Figure 8:**
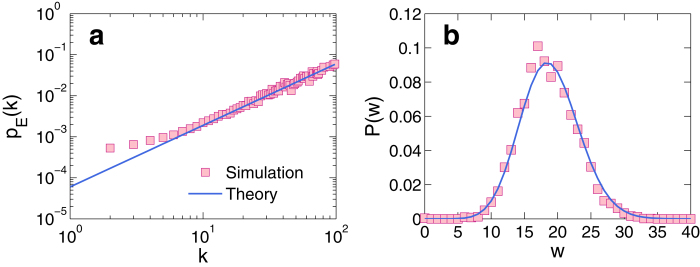
Normalized effective betweenness and free flux distribution function. For a single layer network of size *N* = 1000, (**a**) normalized effective betweenness of a node of degree *k*, *p*_E_, versus *k*, from simulation (squares) and theory (line). The simulation results are obtained from 20 network realizations. (**b**) The free flux distribution function *P*(*w*) for a node of degree 75 versus *w* obtained from simulation (squares) and theory (line), where the distribution function is meaningful only when the system is set to be free of extreme events, i.e., each node is set to have infinite capacity so that packets can travel freely via the shortest path towards their destinations. This way the theoretical probability for the node to be visited can be calculated.

According to Eq. (10), the effective betweenness *B*_E_ of each node can be calculated from information about the network topology, and the probability of a node to be visited (the normalized effective betweenness), *p*_E_, can be obtained from Eq. (11). Through extensive simulations, we find that the effective betweenness under the condition that the contribution of packets is inversely proportional to the path length exhibits a power-law scaling with the degree. The empirical relationship between *p*_E_ and degree *k* is verified by comparing the averaged 
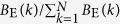
 values corresponding to each *k* value obtained via the topology information and estimated from 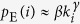, as shown in Fig. 8(a). We see that, except the nodes with very low connectivity, the *P*_E_ value obtained directly from the network topology matches that obtained from the empirical relationship (the error bars are essentially invisible within the scale of the figure).

In our theory, the assumption that packets moving according to the shortest-path routing scheme can be regarded as equivalent to random walks with node-visiting probability proportional to the effective betweenness can be justified through the flux distribution function. As shown in Fig. 8(b), for a typical hub node, the flux distribution function obtained from theory, i.e, the binomial distribution in Eq. (6), correctly fits the statistics obtained from simulation. For certain nodes the theory and simulation results may not match well, but the theoretically predicted and numerically obtain peaks typically have substantial overlaps. Further calculations show that the flux distribution functions obtained from the conventional betweenness centrality deviate from the simulation systematically with large errors, as there is little overlap between the peaks obtained from theory and numerics. This provides further justification for the necessity to instigate the effective betweenness.

## Additional Information

**How to cite this article**: Chen, Y.-Z. *et al.* Extreme events in multilayer, interdependent complex networks and control. *Sci. Rep.* **5**, 17277; doi: 10.1038/srep17277 (2015).
